# Transcriptomic study of the role of *MeFtsZ2-1* in pigment accumulation in cassava leaves

**DOI:** 10.1186/s12864-024-10165-w

**Published:** 2024-05-07

**Authors:** Yuwei Zang, Kunlin Wu, Liangwang Liu, Fangfang Ran, Changyi Wang, Shuwen Wu, Dayong Wang, Jianchun Guo, Yi Min

**Affiliations:** 1 Department of Biosciences, School of Life and Health, Hainan University, Haikou, Hainan 570228 China; 2 Laboratory of Biopharmaceuticals and Molecular Pharmacology, School of Pharmaceutical Sciences and Key Laboratory of Tropical Biological Resources of the Ministry of Education of China, Hainan University, Haikou, Hainan 570228 China; 3 Institute of Tropical Biotechnology, Sanya Institute, Chinese Academy of Tropical Agricultural Sciences, Chinese Academy of Tropical Agricultural Sciences, Sanya, Hainan 572000 China

**Keywords:** *MeFtsZ2-1*, Cassava, Physiology, Transcriptome, Pigment accumulation

## Abstract

*MeFtsZ2-1* is a key gene for plant plastid division, but the mechanism by which *MeFtsZ2-1* affects pigment accumulation in cassava (*Manihot esculenta* Crantz) through plastids remains unclear. We found that *MeFtsZ2-1* overexpression in cassava (OE) exhibited darker colors of leaves, with increased levels of anthocyanins and carotenoids. Further observation via Transmission Electron Microscopy (TEM) revealed no apparent defects in chloroplast structure but an increase in the number of plastoglobule in OE leaves. RNA-seq results showed 1582 differentially expressed genes (DEGs) in leaves of OE. KEGG pathway analysis indicated that these DEGs were enriched in pathways related to flavonoid, anthocyanin, and carotenoid biosynthesis. This study reveals the role of *MeFtsZ2-1* in cassava pigment accumulation from a physiological and transcriptomic perspective, providing a theoretical basis for improving cassava quality.

**Supplementary Information:**

The online version contains supplementary material available at 10.1186/s12864-024-10165-w.

## Introduction

Cassava (*Manihot esculenta* Crantz), belonging to the Euphorbiaceae family, is native to the southern Amazon basin and is widely cultivated in tropical and subtropical regions, ranking as the world’s sixth-largest crop [1]. Despite the starch-rich storage roots, frequent consumption can lead to hidden hunger due to low levels of other nutrients [2]. In recent years, leaves have gained recognition as a substitute source of protein and micronutrients in sub-Saharan Africa and parts of Asia [3]. Research indicates that cassava tuberous roots with different flesh colors exhibit varying nutritional profiles, with darker-colored cassava potentially harboring elevated levels of secondary metabolites and nutrients, particularly carotenoids and flavonoids [4–6]. Therefore, augmenting the content of carotenoids and anthocyanins in cassava tuberous roots and leaves holds the potential to enhance the nutritional and health value of cassava.

In plants, chlorophyll, carotenoids, and anthocyanins are regulated by various factors, including environmental conditions and plant hormones [7]. For instance, exposure to light increases carotenoid levels in Arabidopsis *(Arabidopsis thaliana)*. Plant hormones such as jasmonic acid (JA), salicylic acid (SA), and ethylene can modulate pigment synthesis, promoting the accumulation of anthocyanins, chlorophyll, and carotenoids [8, 9]. Conversely, high concentrations of abscisic acid (ABA) reduce chlorophyll content, while low concentrations enhance its accumulation [10].

As a place for the synthesis and storage of various pigments, plastids play a vital role in the accumulation of pigments [11]. During fruit ripening, carotenoid levels increase with changes in the size and shape of plastoglobules in plastids [12]. The regulation of plastid division impacts not only plastid numbers but also the synthesis and accumulation of substances on plastids [13, 14]. Plastid division is primarily regulated by several key genes, including *cell division protein FtsZ homolog 1* (*FtsZ1*), *cell division protein FtsZ homolog 2 − 1* (*FtsZ2-1*), *dynamin-like protein ARC5* (*ARC5*), *protein accumulation and replication of chloroplasts 6* (*ARC6*), *plastid division protein PDV1* (*PDV1*), *plastid division protein PDV2* (*PDV2*), and other genes [15, 16]. The assembly of *FtsZ1* and *FtsZ2-1* into the inner ring of plastid division is a key step in plastid division, and *FtsZ2-1* can maintain the stability of the inner ring structure [17]. In *Arabidopsis thaliana*, the disorder of FtsZ protein expression leads to abnormal chloroplast division, resulting in a decrease in the number and an increase in the volume of chloroplasts [18, 19]. This indicates that previous studies primarily focused on the role of FtsZ proteins in plastid division, with limited research on their impact on secondary metabolism.

In this study, we wanted to understand how *MeFtsZ2-1* affects cassava pigment accumulation. We compared the color, chloroplast ultrastructure, chlorophyll, carotenoid and anthocyanin content of *MeFtsZ2-1* overexpression cassava (OE) and wild-type cassava (WT). Subsequently, transcriptome data were used to analyze the related pathways affected by *MeFtsZ2-1*. This study provides a molecular perspective for the regulation of pigment accumulation in cassava, thus laying a foundation for the improvement of cassava germplasm.

## Results

### Molecular identification and phenotypic analysis of cassava

**Fig. 1:**
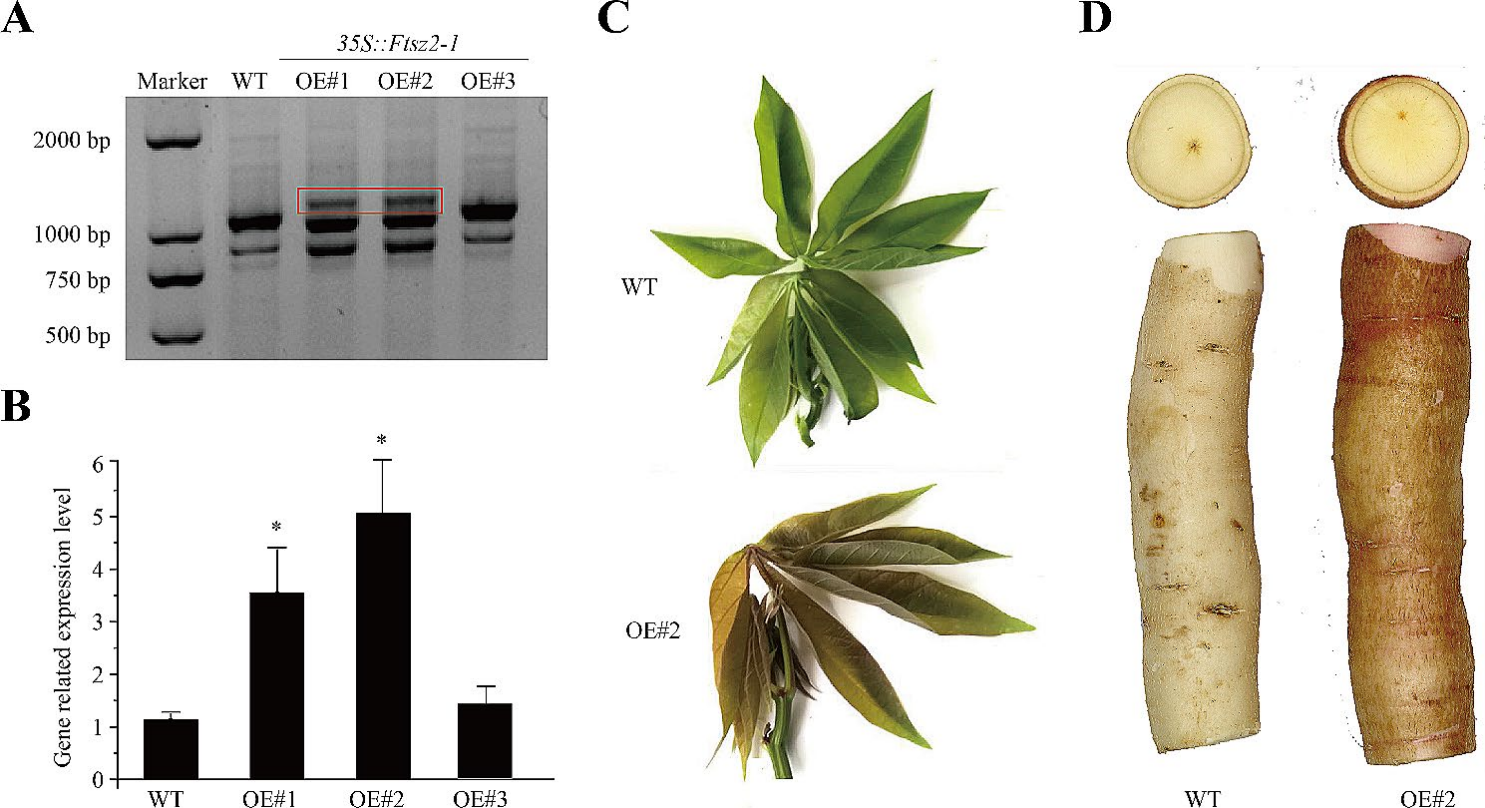
Molecular identification and phenotypes of WT and OE cassava. (**A**) RT-PCR analysis of *MeFtsZ2-1* expression in OE plants using specific primers for the *MeFtsZ2-* gene. The red box is 35::*MeFtsZ2-1* PCR products; (**B**) qRT-PCR analysis of transformants using quantified primers for *MeFtsZ2-1*; (**C**) Leaf phenotype and (**D**) tuberous root tuber phenotype. WT, wild-type cassava. OE#1, OE#2 and OE#3, *MeFtsZ2-1* overexpression cassava

The results revealed that the transcription levels of the *MeFtsZ2-1* gene were higher in OE compared to WT (Fig. 1A-B and Supplement Figure S1). According to the results of PCR and quantitative real-time PCR (qRT-PCR), we selected OE#2 as the follow-up experimental material. In order to observe the effect of MeFtsZ2-1 on the color of cassava, we compared the fourth leaves and tuberous roots of 8-month-old OE and WT. In OE, the color of leaves and petioles was reddish brown, the color of the epidermis of the tuberous roots was yellowish brown, and a layer of pink flesh was visible under the epidermis of the tuberous roots (Fig. 1C-D and Supplement Figure S2). The color of WT leaves and petioles remained green, the color of the tuberous roots epidermis was white, and the tuberous roots epidermis was a layer of white flesh (Fig. 1C-D and Supplement Figure S2). These results indicate that there is a correlation between the *MeFtsZ2-1* gene and the cassava color phenotype.

### Ultrastructural observation of chloroplasts in OE

**Fig. 2:**
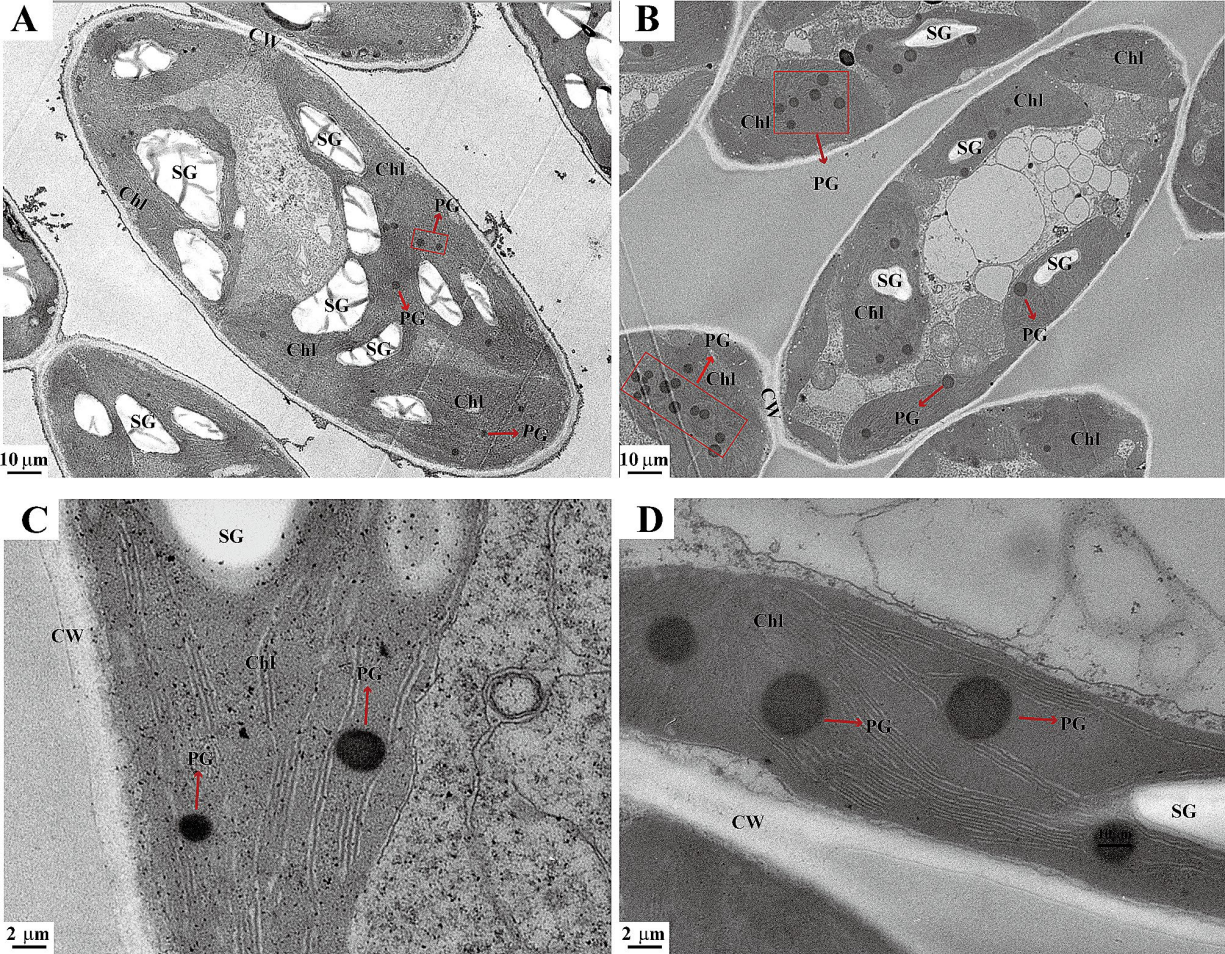
Comparison of plastid ultrastructure in Leaves of WT and OE cassava. (**A** and **C**). WT cassava leaves. (**B** and **D**). OE cassava leaves. Chl: Chloroplasts; CW: cell wall; PG: Plastoglobuli; SG: Starch grain. WT, wild-type cassava. OE, *MeFtsZ2-1* overexpression cassava

The results of electron microscopy revealed a decrease in the volume of chloroplasts within OE leaves, however, the structure remained unaltered (Fig. 2A-B). Within the OE chloroplast, the volume of plastoglobules experienced an increase, while the volume and number of starch granules decreased (Fig. 2C-D). These results suggest that *MeFtsZ2-1* induced alterations in cassava color might be associated with modifications in material accumulation sites.

### Analysis of chlorophylls, carotenoids and anthocyanins content

**Fig. 3:**
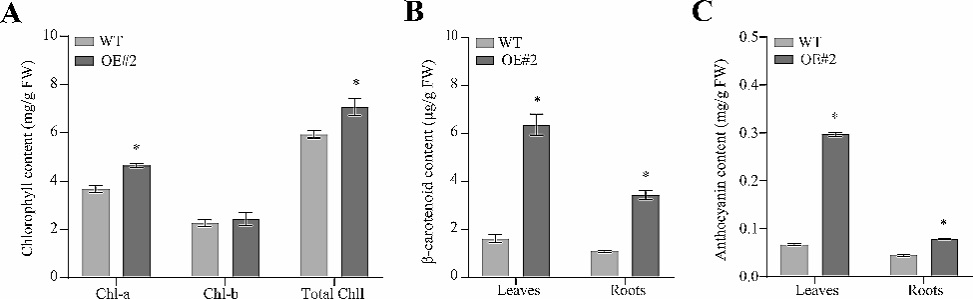
Pigment content analysis of WT and OE cassava; (**A**) Chlorophyll content; (**B**) Anthocyanin content; (**C**) Carotenoid content. WT, wild-type cassava. OE, *MeFtsZ2-1* overexpression cassava. Error bars represent the SD of the mean values (*: *p* < 0.05). Each column represents the mean of three independent measurements

In order to clarify the effect of *MeFtsZ2-1* on the color change of cassava, the contents of chlorophylls, carotenoids, and anthocyanins in WT and OE were detected. The results showed that compared with WT leaves (WTL), the total chlorophyll content and chlorophyll a content of OE leaves (OEL) increased significantly, and there was no significant difference in chlorophyll b content (Fig. 3A). The content of β-carotene and anthocyanin in OEL was significantly higher than that in WTL (Fig. 3B-C). Compared with WT tuberous roots (WTR), the total chlorophyll content and chlorophyll A content of OE tuberous roots (OER) increased significantly (Fig. 3B-C). Overexpression of *MeFtsZ2-1* in cassava led to increased levels of chlorophylls, carotenoids, and anthocyanins, indicating that the overexpression of the *MeFtsZ2-1* gene might impact chlorophyll, carotenoids, and anthocyanins synthesis and accumulation in cassava tissues.

### Identification and analysis of differentially expressed genes (DEGs)

**Fig. 4:**
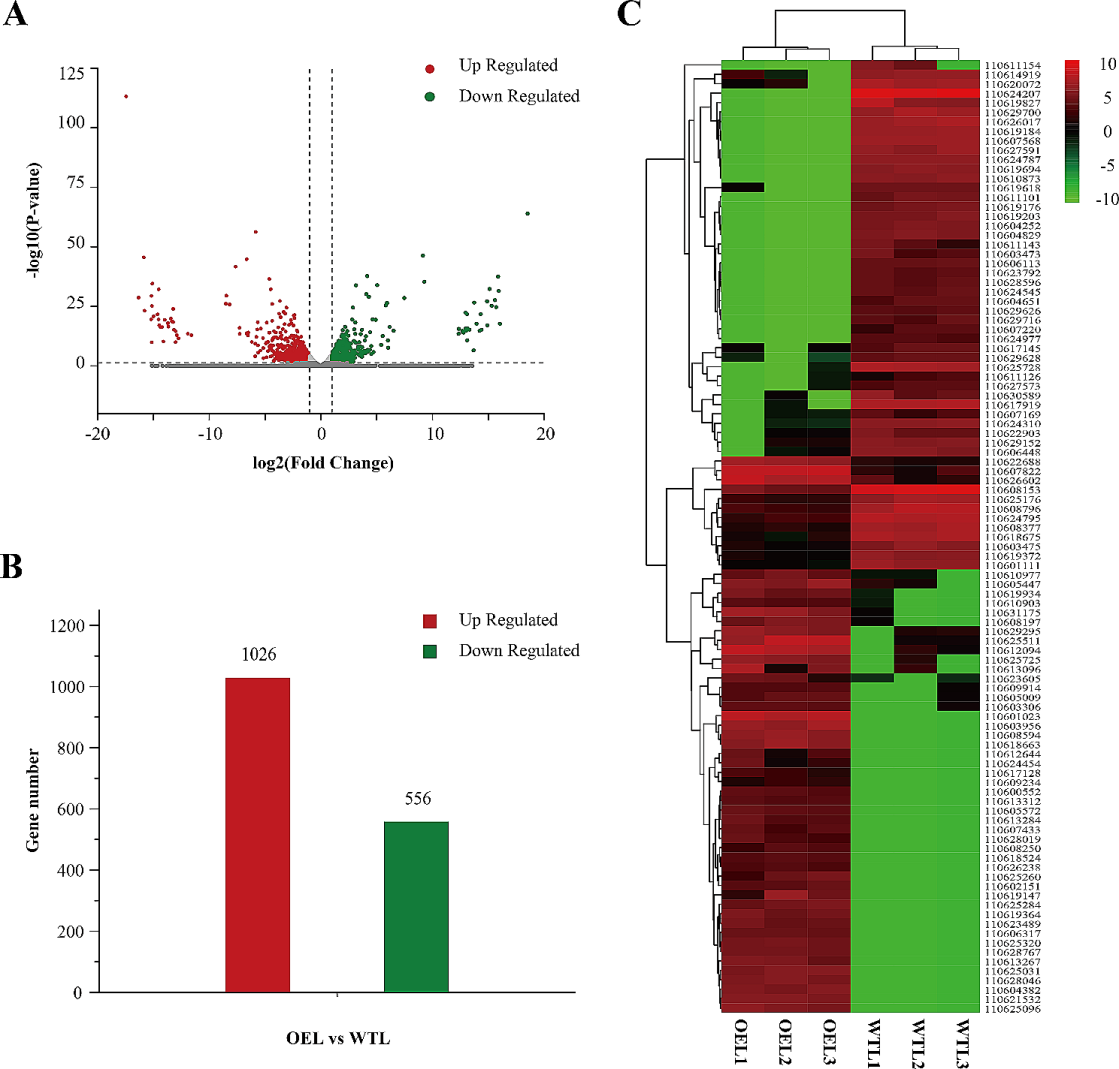
RNA-seq analysis of differentially expressed transcripts in WT and OE leave. (**A**) Volcano plot of the number of DEGs in leaves. (**B**) Bar graph of the number of DEGs in leaves. (**C**) Heatmap of the DEGs in leaves. WT, wild-type cassava. OE, *MeFtsZ2-1* overexpression cassava

Two transcriptomic comparisons were performed to identify differentially expressed genes (DEGs) between the OEL and WTL (Supplement Table S1 and S2). A total of 1582 DEGs were identified in the OEL, with 1026 genes upregulated and 556 genes downregulated, when compared to the WTL (Fig. 4A-B). Figure 4C shows the expression of DEGs in the transcriptome of cassava leaves. DEGs exhibiting overlapping expression in leaves of OE could play crucial roles in cassava growth and development.

**Fig. 5:**
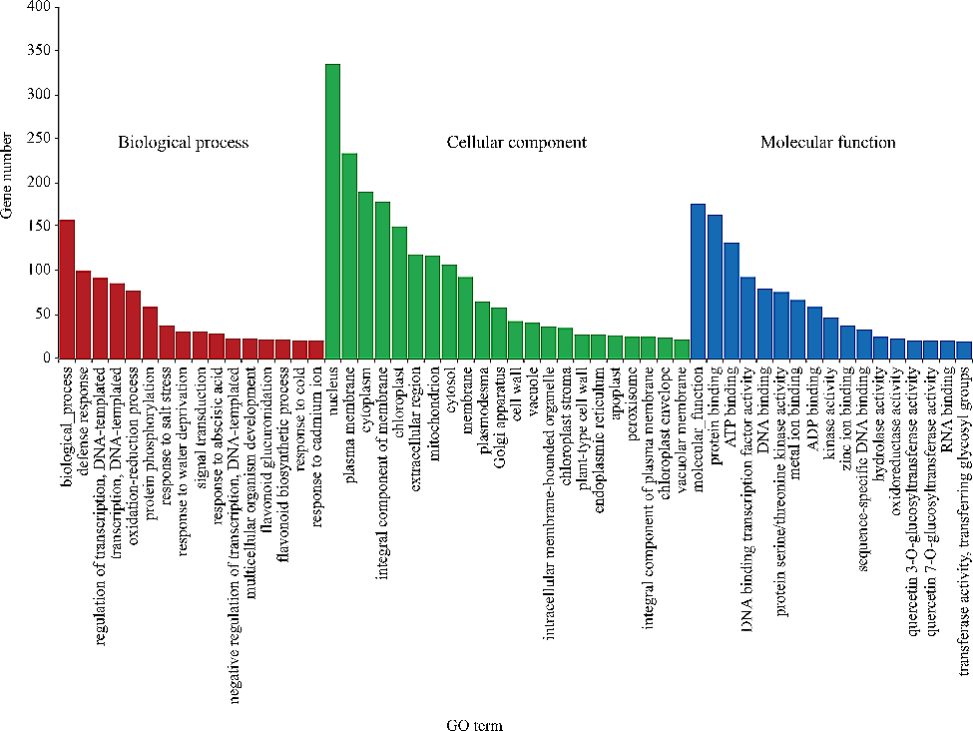
Gene ontology (GO) enrichment classification of differentially expressed genes in cassava leaves. All annotated unigenes were divided into three functional GO categories: biological process, cellular component and molecular function

GO classification analysis was performed on the DEGs of OEL/WTL (Fig. 5). In the OEL/WTL comparison, a majority of DEGs were categorized under defense response clusters in relation to biological processes. DEGs associated with cellular components were linked to cell nucleus, plasma membrane, cytoplasm, membrane components, and chloroplasts. The functionality of DEGs was primarily related to protein binding, ATP binding, and DNA binding transcription factor activity.

KEGG pathway analysis showed that the main pathways of OEL/WTL included glycolysis, gluconeogenesis, citric acid metabolism, fatty acid degradation, glycolysis/gluconeogenesis, phenylpropionic acid biosynthesis, anthocyanin biosynthesis and carotenoid metabolism (Supplementary Figure S3A).

### Identification of genes involved in chlorophylls, carotenoids and anthocyanins biosynthesis pathways

**Fig. 6:**
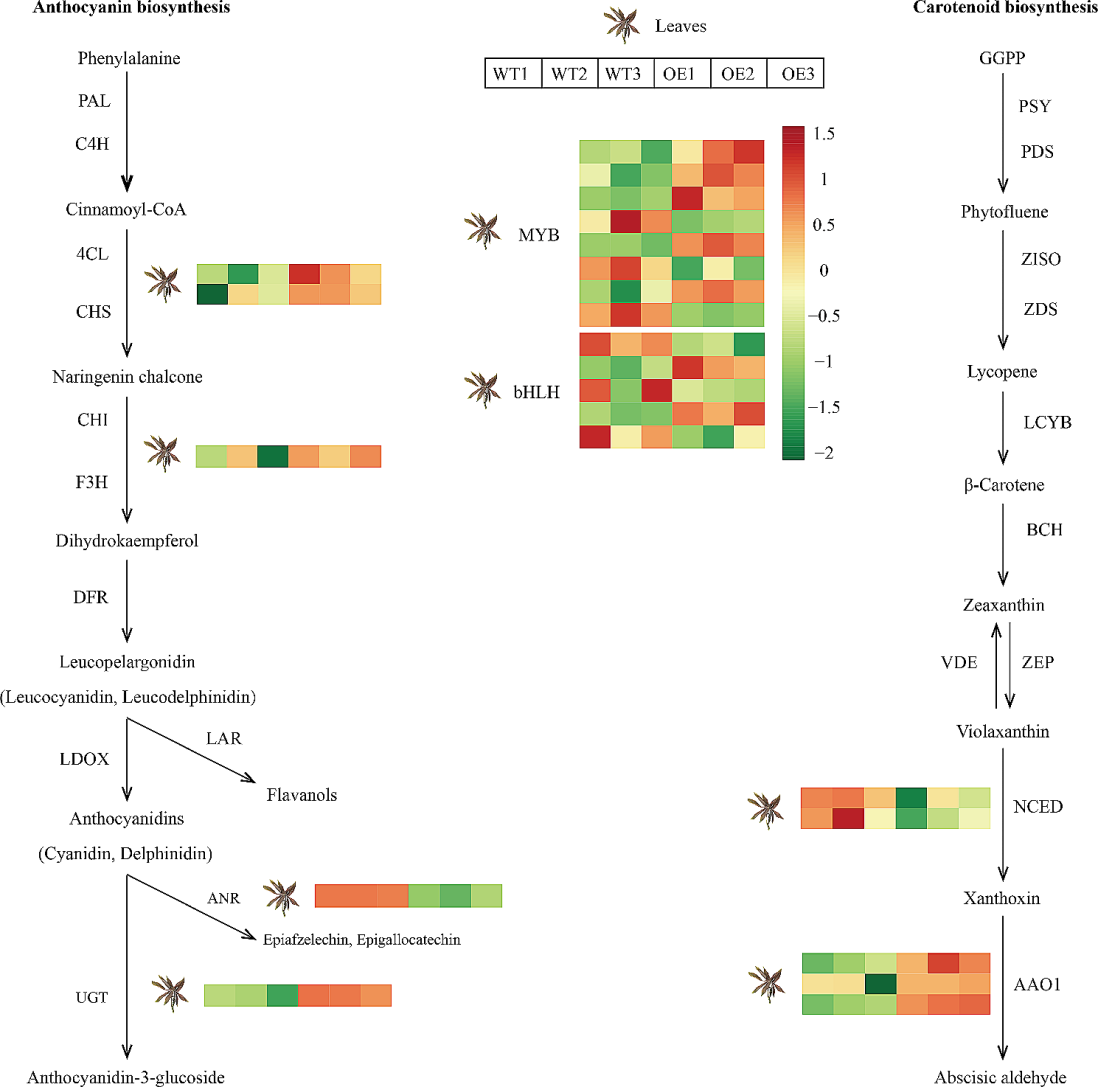
Heatmaps of DEGs related to anthocyanidin and β-carotenoid biosynthesis in the WT and OE cassava. *PAL, phenylalanine ammonia lyase*; *C4H, cinnamate 4-hydroxylase*; *4CL, 4coumaroyl CoA ligase*; *CHS, chalcone synthase*; *CHI, chalcone isomerase*; *F3H, flavanone 3-hydroxylase*; *DFR, dihydroflavonol-4-reductase*; *LDOX, leucoanthocyanidin dioxygenase*; *ANR, Anthocyanidin reductase*; *LAR, leucocyanidin reductase*; *UGT, UDP glycosyltransferase*; *PSY, phytoene synthase*; *PDS, phytoene desaturase*; *Z-ISO, ζ-carotene isomerase*; *ZDS, ζ-carotene desaturase*; *LYCE, lycopene epsilon-cyclase*; *BCH, β-carotene hydrolase*; *ZEP, zeaxanthin epoxidase*; *VDE, violaxanthin de-epoxidase*; *NCED, 9-cis-epoxycarotenoid dioxygenase*; *ABA2, xanthoxin dehydrogenase*; Significant changes in gene expression levels are shown by the intensities of colors expressed in log2 values. WT, wild-type cassava. OE, *MeFtsZ2-1* overexpression cassava

A comparison between OEL/WTL revealed a total of 26 differentially expressed genes (DEGs) within the chlorophylls, carotenoids and anthocyanins synthesis pathway (Supplement Table S3). We identified 6 DEGs associated with chlorophylls biosynthesis and metabolism pathways. Among these, *transcription factor GLABRA 3 (GL3)*, *Protein STAY-GREEN homolog (SGR)*, *ABC transporter C family member 3 (ABCC3)*, *transcription factor PIF1 (PIF1)*, and *putative U-box domain-containing protein 42 (PUB42)* exhibited higher expression levels in OEL. We identified six DEGs related to carotenoids biosynthesis in OEL, in which the expression of *indole-3-acetaldehyde oxidase (AAO1)* was up-regulated, *9-cis-epoxycarotenoid dioxygenase NCED3 (NCED3)* was down-regulated (Fig. 6). The expression levels of genes related to the anthocyanins synthesis pathway, such as *4-coumarate–CoA ligase (4CL)*, *Naringenin,2-oxoglutarate 3-dioxygenase* (*F3H)*, and *UDP-glycosyltransferases* (*UGT)*, were significantly increased in OEL (Fig. 6).

### Identification of transcription factors (TFs)

In the transcriptomic databases of OEL and WTL, 49 differentially expressed genes (DEGs) encoding transcription factors (TFs) were identified and classified into 11 different TF families (Supplementary Figure S3B). The WRKY family had the highest number of DEGs (10 genes), followed by MYB (9 genes), bHLH (8 genes), AP2-ERF (5 genes), and TFIID (2 genes). These results indicate that the MeFtsZ2-1 gene can have multiple regulatory mechanisms in leaves.

### DEGs associated with hormone signaling pathways

In OE, a total of 41 differentially expressed genes (DEGs) related to plant hormones were identified in leaves, respectively (Supplement Table S4 and Figure S4). Compared to the WT, within the auxin signaling pathway, *auxin-induced proteins (AUX)*, *auxin-responsive protein genes (SAUR/IAA)*, and *auxin transporter protein (LAX)* exhibited higher expression levels in the OEL. However, *indole-3-acetic acid-amido synthetases (GH3.1/GH3.9)* and *auxin response factor (ARF)* showed decreased expression in OEL. In the salicylic acid signaling pathway, the expression of *transcription factor TGA (TGA1)* and *pathogenesis-related protein 1 (PRB1)* was upregulated in OE. Within the gibberellin signaling pathway, *chitin-inducible gibberellin-responsive protein 1 (CIGR1)* was significantly downregulated in OEL, while *scarecrow-like transcription factor PAT1 (PAT1)* and *transcription factor PIF1 (PIF1)* were markedly upregulated. In the ABA signaling pathway, *abscisic acid receptors (PYL)* and *serine/threonine-protein kinase (SRK2A)* were upregulated, whereas *probable protein phosphatase 2 C (PP2C51)*, *transcription factor HHO (HHO2)*, and *histidine kinase 5 (AHK5)* were downregulated.

### Quantitative real-time PCR (qRT-PCR) analysis

**Fig. 7:**
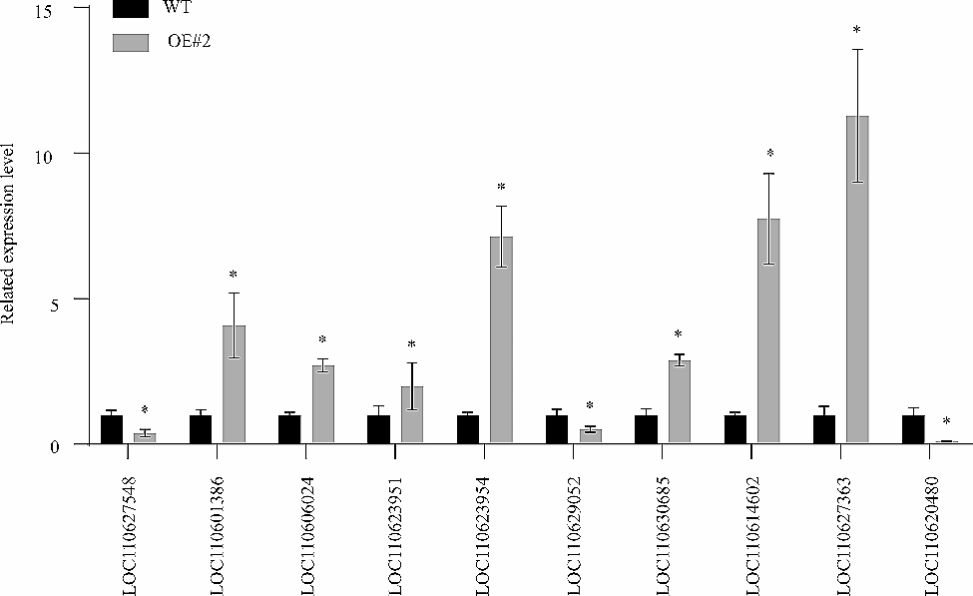
Ten candidate genes were randomly selected from leaves for qRT-PCR verification. All measurements were replicated three times. Vertical bars represent S.E. of the mean (*n* = 3). Data are mean ± SE of three individual experiments, each performed in triplicate (*: *p* < 0.05). WT, wild-type cassava. OE, *MeFtsZ2-1* overexpression cassava

Ten randomly selected DEGs from both leaves and tuberous roots were subjected to qRT-PCR analysis to validate the reliability of the transcriptome sequencing data (Supplement Table S5). The qRT-PCR results were consistent with the transcriptomic data, indicating the high reliability of the transcriptome data (Fig. 7).

## Discussion

The increase in substances such as anthocyanins and carotenoids in crops is beneficial not only for human health but also for enhancing survival capabilities in stressful environments [20]. Currently, most studies focus on elevating the levels of carotenoids and anthocyanins in crops by manipulating specific genes involved in their biosynthesis. However, research on increasing the levels of carotenoids and anthocyanins by altering their synthesis sites has not been reported.

Plastids are membrane-bound organelles responsible for storing substances such as chlorophylls and carotenoids in plant cells [21]. Severe abnormalities in plastid division have been observed in mutants like *ARC5* and *ARC6*, leading to a decrease in chlorophyll content within the chloroplasts [22, 23]. Manipulating plastid division-related genes such as *ARC3* and *PDV* in *Arabidopsis thaliana* can alter the number of plastids, subsequently affecting carotenoid levels [14]. Plastoglobule are the place where carotenoids accumulate [24]. Stimulation to increase the number of plastoglobule in melon (*Cucumis melo*) can increase the accumulation of carotenoids [25]. In our study, we observed that overexpression of *MeFtsZ2-1* resulted in elevated chlorophyll and carotenoid levels in cassava leaves. Moreover, there was a notable increase in the number of plastid stromules in OEL, implying that the overexpression of *MeFtsZ2-1* may augment chlorophylls and carotenoids content by expanding the storage capacity within plastids. Additionally, the content of chlorophylls and carotenoids in plants is influenced by their synthesis and degradation rates. Transcription factors known as *phy-interacting factors (PIFs)*, members of the basic helix-loop-helix family, can regulate the expression of genes such as *protochlorophyllide oxidoreductase (POR)*, *ferrochelatase (FeChII)*, and *heme oxygenase (HO3)*, which control chlorophyll synthesis [26]. In our study, we observed a significant upregulation of *PIF1* in OEL, suggesting a potential role in chlorophyll synthesis regulation. The *NCED* gene, belonging to the carotenoid cleavage oxygenase (CCO) family, negatively regulates carotenoid synthesis in various species by inhibiting carotenoid degradation [27]. Transcriptome data revealed a significant decrease in *NCED* expression in carotenoid pathways in OEL, suggesting that overexpression of *MeFtsZ2-1* may not only affect storage space but also the expression of genes involved in chlorophyll and carotenoid metabolism pathways.

In many plants such as sweet potatoes *(Ipomoea batatas L.)*, strawberries *(Fragaria×ananassa)*, pears *(Pyrus ussuriensis)*, and sweet cherries *(Prunus avium L.)*, changes in plant color phenotypes are often associated with the synthesis, transport, and accumulation of anthocyanins [28–31]. In this study, we observed that overexpression of *MeFtsZ2-1* led to an increase in anthocyanins content within the plant, resulting in darker coloration of leaves. RNA-seq comparative analysis revealed significant upregulation of the expression of genes such as *4CL*, *F3H*, *CHS*, and *UGT* in the OE. Previous research has shown that overexpression of *4CL* and *F3H* in plants can promote the accumulation of anthocyanins [32]. Taken together, after the overexpression of the *MeFtsZ2-1* gene, the expression of genes controlling anthocyanin synthesis increased, and the expression of genes regulating anthocyanin degradation decreased, leading to the accumulation of anthocyanins in the OEL. Furthermore, various transcription factors can regulate pigment metabolism by modulating the expression of genes involved in anthocyanin and carotenoid biosynthesis, thereby influencing anthocyanin and carotenoid content and altering plant flower color [33]. In our study, we observed the significant upregulation of MYB and bHLH family genes in OE. These upregulated transcription factors are likely responsible for the differential expression of genes involved in the pigment metabolic pathways. Based on these results, we speculate that these TFs and differentially expressed genes play a crucial role in anthocyanin metabolism in OE. However, further research is needed to elucidate how the *MeFtsZ2-1* gene induces color changes.

The biosynthesis pathways of carotenoids and anthocyanins in plants are regulated by various plant hormones, each of which has distinct effects on their synthesis. Exogenous application of auxin and ethylene increases carotenoid content in Chlorella *(Chlorella sp. BR2)* and loquat *(Eriobotrya japonica Lindl cv. Algerie)*, and ethylene also enhances the expression of carotenoid biosynthesis genes and transcription factors [34–36]. The application of abscisic acid in tea *(Camellia sinensis)* flowers can significantly reduce the content of carotenoids [37]. Regarding the impact on the anthocyanin biosynthesis pathway, the loss of function of *auxin response factors (ARFs)* leads to a significant decrease in anthocyanin content in Arabidopsis [38]. In red-skinned pears *(Pyrus pyrifolia Nakai)*, *ethylene response factors (ERFs)* can both positively regulate anthocyanin biosynthesis and negatively inhibit fruit anthocyanin biosynthesis [39]. Treatment with ABA in grapes *(Vitis vinifera L.)* reduces the activity of *LAR* and *ANR* and inhibits the expression of related genes, affecting the anthocyanin biosynthesis pathway [40]. In this study, we observed that overexpression of *MeFtsZ2-1* increased the content of carotenoids and anthocyanins in cassava leaves. Therefore, we speculate that the color changes in cassava leaves are regulated by various hormones. However, the specific regulatory mechanism needs further study.

In summary, the overexpression of *MeFtsZ2-1* significantly influences the morphology and quantity of chloroplasts in cassava, while having no significant impact on their structure. Moreover, *MeFtsZ2-1* promotes the accumulation of chlorophyll, carotenoids, and anthocyanins by modulating the biosynthesis of pigments and hormones, as well as genes related to signal transduction. These findings hold promise for enhancing cassava quality and developing nutrient-enriched cassava plants.

## Materials and methods

### Plant materials and growth conditions

In this experiment, South China No. 8 (SC8) cassava and *MeFtsZ2-1* overexpression cassava variety, developed at the School of Life Sciences of Hainan University, were employed as experimental materials. The *MeFtsZ2-1* gene sequence and cloning method were provided by Mengting Geng [41]. The method of *MeFtsZ2-1* transgenic cassava was performed according to the method of Yajie Wang [42]. We extracted cassava DNA and RNA according to the DNA extraction kit (Vazyme, Nanjin, China) and the RNA extraction kit (Vazyme, Nanjin, China). The extracted cassava DNA and RNA were used as templates for RT-PCR and RT-qPCR analysis. According to the results of PCR and quantitative real-time PCR, we selected OE#2 as the follow-up experimental material. Leaves (the fourth leaves under the apical buds of 8-month-old cassava) and tuberous roots (8-month-old cassava) were harvested from the tropical crop experimental base of Hainan University (30°52’N 121°54’E), with each sample having three biological replicates. Following collection, each sample was divided into three equal parts. One portion of the samples was rapidly frozen using liquid nitrogen and stored at -80 °C for subsequent RNA extraction and transcriptome sequencing. The second portion of leaves was fixed with 2.5% glutaraldehyde, while the third portion, constituting fresh samples, was used to measure various physiological indices.

### Transmission electron microscopy

Leaves were initially cut into 1 mm cubes, excised, and promptly fixed using 2.5% (v/v) glutaraldehyde (containing 0.05 M sodium cacodylate/HCl, pH = 7) for 2 h at room temperature, followed by overnight incubation at 4 °C. After rinsing with buffer, the samples were immersed in 1% (w/v) buffered osmium tetroxide at 22 °C for 5 h. Subsequently, prior to embedding in resin, the samples underwent rinsing in distilled water and dehydration in ethanol. Thin sections were stained with uranyl acetate and lead citrate for 1 h and 15 min, respectively. Stained sections were examined at 100 kV using a TEM (JEOL JEM-1200EXII UK, Hertfordshire, UK), and images were captured.

### Determination of chlorophyll content

Lyophilized leaves (0.3 g) were immersed in 5 mL of methanol containing 0.1% HCl and stored in darkness overnight. The resulting supernatant was subjected to centrifugation at 5000 rpm for 10 min, and the absorbance was subsequently measured at 525 nm using a UV spectrophotometer (UV-1800PC, Shanghai). Anthocyanin content was quantified based on a standard curve generated with cyanidin-3-O-glucoside.

Fresh leaves were sliced and individually weighed. Subsequently, 1 g of leaf material was placed into centrifuge tubes containing 15 mL of 95% ethanol (v/v). During the extraction process, the centrifuge tubes were vigorously shaken multiple times, and the samples were stored in the dark at 4 °C in a refrigerator for 24 h until the leaves became completely colorless. Blank controls were prepared using 95% ethanol. Chlorophyll a content was calculated as follows: Chl a = 13.95A665 nm − 6.88A649 nm. Chl b = 24.96A649 nm − 7.32A665 nm. Chl = (Chl a + Chl b) × V ÷ 1000 W [43].

### Determination of anthocyanin and β-carotene of cassava leaves and tuberous roots by HPLC

Fresh samples of cassava leaves and tuberous roots were homogenized, followed by rapid transfer to pre-cooled centrifuge tubes. The analytical procedure utilized a C30 column (YMC, 4.6 mm × 250 mm, 5 μm). The mobile phase consisted of methanol: MTBE (methyl tert-butyl ether) in an 8:2 (V: V) ratio. An isocratic elution was performed at a flow rate of 0.8 mL/min, with an automatic loading of 20 µL of the extracted sample. The column temperature was maintained at 30 °C, and detection was done at a wavelength of 450 nm. Anthocyanin and β-carotene concentrations were quantified using external standards, with three biological replicates conducted for each compound.

### cDNA library construction and Illumina sequencing

Total RNA from WTL and OEL were extracted using TRIzol reagent (Invitrogen, CA, USA) following the manufacturer’s instructions. The resulting RNA was reverse transcribed into cDNA and subjected to PCR cycles using adaptor primers (Supplement Table S6). PCR amplification was also employed for selective enrichment of specific fragments. Subsequently, the cDNA library was purified using the AMPure XP system (Beckman Coulter, Beverly, USA) and sequenced on an Illumina HiSeq™ 2500 platform.

### Differential expression analysis and functional enrichment

The expression levels of individual transcripts were determined using the transcripts per million reads (TPM) method for the identification of differentially expressed genes (DEGs). Gene abundance was quantified using RSEM (v1.1.12). Differential expression analysis was conducted using DESeq2, with significance set at the Q value less than 0.05. Gene functions were annotated using the GO and KEGG databases.

### RNA-seq results verification by quantitative real-time PCR (qRT-PCR)

For the validation of RNA-seq results, ten DEGs from leaves and tuberous roots were randomly selected for qRT-PCR analysis. The primer sequences for qRT-PCR are provided in Supplement Table S5. Total RNA extraction and cDNA synthesis followed the methods described earlier. qRT-PCR was conducted using a Light Cycler® 96 Instrument (Roche, Shanghai, China), with actin serving as the internal reference. Each gene was analyzed with three biological replicates and three technical replicates.

### Statistical analysis

Data regarding pigment concentrations and the relative expression levels of specific genes were subjected to statistical analysis using SPSS 21.0 software. Results are presented as mean ± SD, and statistical significance was determined with a threshold of *P* < 0.05.

### Electronic supplementary material

Below is the link to the electronic supplementary material.

**Supplementary Material 1: **
**Supplemental Table S1**. Overview of quality control for sequencing data.
**Supplemental Table S2**. Reference genome comparison.
**Supplemental Table S3**. Differentially expressed genes in the chlorophylls, carotenoids and anthocyanins biosynthesis pathway.
**Supplemental Table S4**. Differentially expressed genes in the hormone signaling pathway.
**Supplemental Table S5**. Quantitative Real-Time PCR (qRT-PCR) primer sequences.
**Supplemental Table S6**. Gene specific primers sequences

**Supplementary Material 2: **
**Supplemental Figure S1**. RT-PCR analysis of MeFtsZ2-1 expression in OE plants using specific primers for the MeFtsZ2-1 gene.
**Supplemental Figure S2**. Leaf phenotype and tuberous root tuber phenotype.
**Supplemental Figure S3**. The KEGG enrichment analysis of the differentially expressed genes and transcription factor families between OE and WT.
**Supplemental Figure S4**. Changes in genes involved in the plant hormone signal transduction pathway in cassava leaves

## Data Availability

The single cell transcriptome dataset of the Cassava leaves that was used in the article is available at the Genome Sequence Archive (GSA) database of National Genomics Data Center (NGDC) (https://ngdc.cncb.ac.cn/). The accession number for the dataset are CRA013895, CRA013896, CRA013897, CRA013898, CRA013899 and CRA013900.

## References

[1] Olsen K, Schaal B (2001). Microsatellite variation in cassava (Manihot esculenta, Euphorbiaceae) and its wild relatives: further evidence for a southern amazonian origin of domestication. Am J Bot.

[2] Uchechukwu-Agua AD, Caleb OJ, Opara UL (2015). Postharvest Handling and Storage of Fresh Cassava Root and products: a review. Food Bioprocess Technol.

[3] Leguizamon AJ, Rompato KM, Hoyos RE, Audisio MC. Nutritional evaluation of three varieties of cassava leaves (Manihot esculenta Crantz) grown in Formosa, Argentina. J Food Compos Anal 2021, 101.

[4] Xiao L, Cao S, Shang X, Xie X, Zeng W, Lu L, Kong Q, Yan H (2021). Metabolomic and transcriptomic profiling reveals distinct nutritional properties of cassavas with different flesh colors. Food Chem Mol Sci.

[5] Carvalho LJCB, Agustini MAV, Anderson JV, Vieira EA, de Souza CRB, Chen S, Schaal BA, Silva JP. Natural variation in expression of genes associated with carotenoid biosynthesis and accumulation in cassava (< i > Manihot esculenta Crantz) storage root. BMC Plant Biol 2016, 16. 10.1186/s12870-016-0826-0 PMC4902922 27286876

[6] Ayetigbo O, Latif S, Abass A, Mueller J. Comparing characteristics of Root, Flour and Starch of Biofortified Yellow-Flesh and White-Flesh Cassava variants, and sustainability considerations: a review. Sustainability 2018, 10(9).

[7] Solfanelli C, Poggi A, Loreti E, Alpi A, Perata P (2006). Sucrose-specific induction of the anthocyanin biosynthetic pathway in Arabidopsis. Plant Physiol.

[8] Malik ANA, Uddain J, Chin CK, Chew BL, Subramaniam S. Elicitation of protocorm-like bodies (PLBs) of < i > Dendrobium ‘Sabin Blue’ using methyl jasmonate, salicylic acid and melatonin for < i > in> < i > vitro production of anthocyanin. *Phytochemistry Letters* 2021, 43:60–64.

[9] Chung M-Y, Vrebalov J, Alba R, Lee J, McQuinn R, Chung J-D, Klein P, Giovannoni J (2010). A tomato (< i > Solanum lycopersicum) < i > APETALA2/ERF gene, <i > SlAP2a, is a negative regulator of fruit ripening</i >. Plant J.

[10] Liu Y, Chen X, Wang X, Fang Y, Zhang Y, Huang M, Zhao H (2019). The influence of different plant hormones on biomass and starch accumulation of duckweed: a renewable feedstock for bioethanol production. Renewable Energy.

[11] Chu C-C, Swamy K, Li H-m (2020). Tissue-specific regulation of plastid protein import via transit-peptide motifs < SUP > OPEN. Plant Cell.

[12] van Wijk KJ, Kessler F. Plastoglobuli: Plastid Microcompartments with Integrated functions in Metabolism, Plastid Developmental transitions, and environmental adaptation. Annu Rev Plant Biol, *Vol 68* 2017, 68:253–89. 10.1146/annurev-arplant-043015-111737 28125283

[13] Egea I, Barsan C, Bian W, Purgatto E, Latche A, Chervin C, Bouzayen M, Pech J-C (2010). Chromoplast differentiation: current status and perspectives. Plant Cell Physiol.

[14] Sun T, Yuan H, Chen C, Kadirjan-Kalbach DK, Mazourek M, Osteryoung KW, Li L (2020). OR < SUP > his, a natural variant of OR, specifically interacts with Plastid Division factor ARC3 to regulate Chromoplast Number and Carotenoid Accumulation. Mol Plant.

[15] Smith AG, Johnson CB, Vitha S, Holzenburg A (2011). Oligomerization of plant FtsZ1 and FtsZ2 plastid division proteins. Arch Biochem Biophys.

[16] Sung MW, Shaik R, TerBush AD, Osteryoung KW, Vitha S, Holzenburg A (2018). The chloroplast division protein ARC6 acts to inhibit disassembly of GDP-bound FtsZ2. J Biol Chem.

[17] Chen C, Cao LY, Yang Y, Porter KJ, Osteryoung KW (2019). ARC3 activation by PARC6 promotes FtsZ-Ring remodeling at the Chloroplast Division Site. Plant Cell.

[18] Porter KJ, Cao L, Chen Y, TerBush AD, Chen C, Erickson HP, Osteryoung KW. The < i > Arabidopsis thaliana Chloroplast division protein FtsZ1 counterbalances FtsZ2 filament stability < i > in vitro. J Biol Chem 2021, 296. 10.1016/j.jbc.2021.100627 PMC8142252 33812992

[19] Stokes KD, McAndrew RS, Figueroa R, Vitha S, Osteryoung KW (2000). Chloroplast division and morphology are differentially affected by overexpression of FtsZ1 and FtsZ2 genes in Arabidopsis. Plant Physiol.

[20] Letif S, Mueller J (2015). Potential of cassava leaves in human nutrition: a review. Trends Food Sci Tech.

[21] de Pater S, Caspers M, Kottenhagen M, Meima H, ter Stege R, de Vetten N (2006). Manipulation of starch granule size distribution in potato tubers by modulation of plastid division. Plant Biotechnol J.

[22] Fujiwara MT, Yasuzawa M, Kojo KH, Niwa Y, Abe T, Yoshida S, Nakano T, Itoh RD. The < i > Arabidopsis arc5 and < i > arc6 mutations differentially affect plastid morphology in pavement and guard cells in the leaf epidermis. PLoS ONE 2018, 13(2). 10.1371/journal.pone.0192380 PMC5821325 29466386

[23] Chen Y, Asano T, Fujiwara MT, Yoshida S, Machida Y, Yoshioka Y (2009). Plant cells without detectable plastids are generated in the < i > crumpled leaf mutant of < i > Arabidopsis thaliana. Plant Cell Physiol.

[24] Rottet S, Devillers J, Glauser G, Douet V, Besagni C, Kessler F (2016). Identification of Plastoglobules as a site of Carotenoid cleavage. Front Plant Sci.

[25] Zhou X, Sun T, Owens L, Yang Y, Fish T, Wrightstone E, Lui A, Yuan H, Chayut N, Burger J (2023). Carotenoid sequestration protein FIBRILLIN participates in CmOR-regulated beta-carotene accumulation in melon. Plant Physiol.

[26] Moon J, Zhu L, Shen H, Huq E (2008). PIF1 directly and indirectly regulates chlorophyll biosynthesis to optimize the greening process in Arabidopsis. Proc Natl Acad Sci USA.

[27] Priya R, Sneha P, Dass JFP, Doss GPC, Manickavasagam M, Siva R. Exploring the codon patterns between < i > CCD and < i > NCED genes among different plant species. Comput Biol Med 2019, 114. 10.1016/j.compbiomed.2019.103449 31568976

[28] Li G, Lin Z, Zhang H, Liu Z, Xu Y, Xu G, Li H, Ji R, Luo W, Qiu Y et al. Anthocyanin Accumulation in the leaves of the Purple Sweet Potato (< i > Ipomoea batatas L.) cultivars. Molecules 2019, 24(20). 10.3390/molecules24203743 PMC6832942 31627373

[29] Song J, Du L, Li L, Kalt W, Palmer LC, Fillmore S, Zhang Y, Zhang Z, Li X (2015). Quantitative changes in proteins responsible for flavonoid and anthocyanin biosynthesis in strawberry fruit at different ripening stages: a targeted quantitative proteomic investigation employing multiple reaction monitoring. J Proteom.

[30] Sun H, Cao X, Wang X, Zhang W, Li W, Wang X, Liu S, Lyu D. RBOH-dependent hydrogen peroxide signaling mediates melatonin-induced anthocyanin biosynthesis in red pear fruit. Plant Sci 2021, 313. 10.1016/j.plantsci.2021.111093 34763877

[31] Kokalj D, Zlatic E, Cigic B, Vidrih R (2019). Postharvest light-emitting diode irradiation of sweet cherries (< i > Prunus avium L.) promotes accumulation of anthocyanins. Postharvest Biol Technol.

[32] Wu X, Zhang S, Liu X, Shang J, Zhang A, Zhu Z, Zha D. Chalcone synthase (CHS) family members analysis from eggplant (< i > Solanum melongena L.) in the flavonoid biosynthetic pathway and expression patterns in response to heat stress. PLoS ONE 2020, 15(4). 10.1371/journal.pone.0226537 PMC7164647 32302307

[33] Wang Y, Zhou L-J, Wang Y, Geng Z, Ding B, Jiang J, Chen S, Chen F. An R2R3-MYB transcription factor CmMYB21 represses anthocyanin biosynthesis in color fading petals of chrysanthemum. Sci Hort 2022, 293.

[34] Alsenani F, Wass TJ, Ma R, Eltanahy E, Netzel ME, Schenk PM (2019). Transcriptome-wide analysis of < i > Chlorella  reveals auxin-induced carotenogenesis pathway in green microalgae. Algal Research-Biomass Biofuels Bioprod.

[35] Alos E, Martinez-Fuentes A, Reig C, Mesejo C, Zacarias L, Agusti M, Rodrigo MJ (2019). Involvement of ethylene in color changes and carotenoid biosynthesis in loquat fruit (< i > Eriobotrya japonica  Lindl. Cv. Algerie). Postharvest Biol Technol.

[36] Xiao X, Shi L, Dong W, Jin S, Liu Q, Chen W, Cao S, Yang Z. Ethylene promotes carotenoid accumulation in peach pulp after harvest. Sci Hort 2022, 304.

[37] Baldermann S, Yang Z, Sakai M, Fleischmann P, Morita A, Todoroki Y, Watanabe N (2013). Influence of exogenously applied abscisic acid on carotenoid content and water uptake in flowers of the tea plant (Camellia sinensis). J Sci Food Agric.

[38] Jiang W, Xia Y, Su X, Pang Y. ARF2 positively regulates flavonols and proanthocyanidins biosynthesis in < i > Arabidopsis thaliana. Planta 2022, 256(2). 10.1007/s00425-022-03936-w 35857143

[39] Sun H, Hu K, Wei S, Yao G, Zhang H (2023). ETHYLENE RESPONSE FACTORS 4.1/4.2 with an EAR motif repress anthocyanin biosynthesis in red-skinned pears. Plant Physiol.

[40] Lacampagne S, Gagne S, Geny L (2010). Involvement of Abscisic Acid in Controlling the Proanthocyanidin Biosynthesis pathway in grape skin: New Elements regarding the regulation of Tannin Composition and Leucoanthocyanidin Reductase (LAR) and Anthocyanidin Reductase (ANR) activities and expression. J Plant Growth Regul.

[41] Geng MT, Min Y, Yao Y, Chen X, Fan J, Yuan S, Wang L, Sun C, Zhang F, Shang L et al. Isolation and characterization of Ftsz genes in Cassava. Genes (Basel) 2017, 8(12). 10.3390/genes8120391 PMC5748709 29244730

[42] Wang YJ, Lu XH, Zhen XH, Yang H, Che YN, Hou JY, Geng MT, Liu J, Hu XW, Li RM et al. A Transformation and Genome Editing System for Cassava Cultivar SC8. *Genes (Basel)* 2022, 13(9). 10.3390/genes13091650 PMC9498335 36140817

[43] Yang W, Lin Y, Xue Y, Mao M, Zhou X, Hu H, Liu J, Feng L, Zhang H, Luo J (2022). Light intensity affects the coloration and structure of chimeric leaves of Ananas comosus var. Bracteatus. Phyton-International J Experimental Bot.

