# Supplementary material for: Transcriptomic study of the role of MeFtsZ2-1 in pigment accumulation in cassava leaves
Source: BMC Genomics. 2024 May 7;25:448. doi: 10.1186/s12864-024-10165-w (PMC11129481; doi:10.1186/s12864-024-10165-w)
Supplement: Supplementary file 2 — Supplementary Material 2: Supplemental Figure S1. RT-PCR analysis of MeFtsZ2-1 expression in OE plants using specific primers for the MeFtsZ2-1 gene. Supplemental Figure S2. Leaf phenotype and tuberous root tuber phenotype. Supplemental Figure S3. The KEGG enrichment analysis of the differentially expressed genes and transcription factor families between OE and WT. Supplemental Figure S4. Changes in genes involved in the plant hormone signal transduction pathway in cassava leaves [file 12864_2024_10165_MOESM2_ESM.docx]

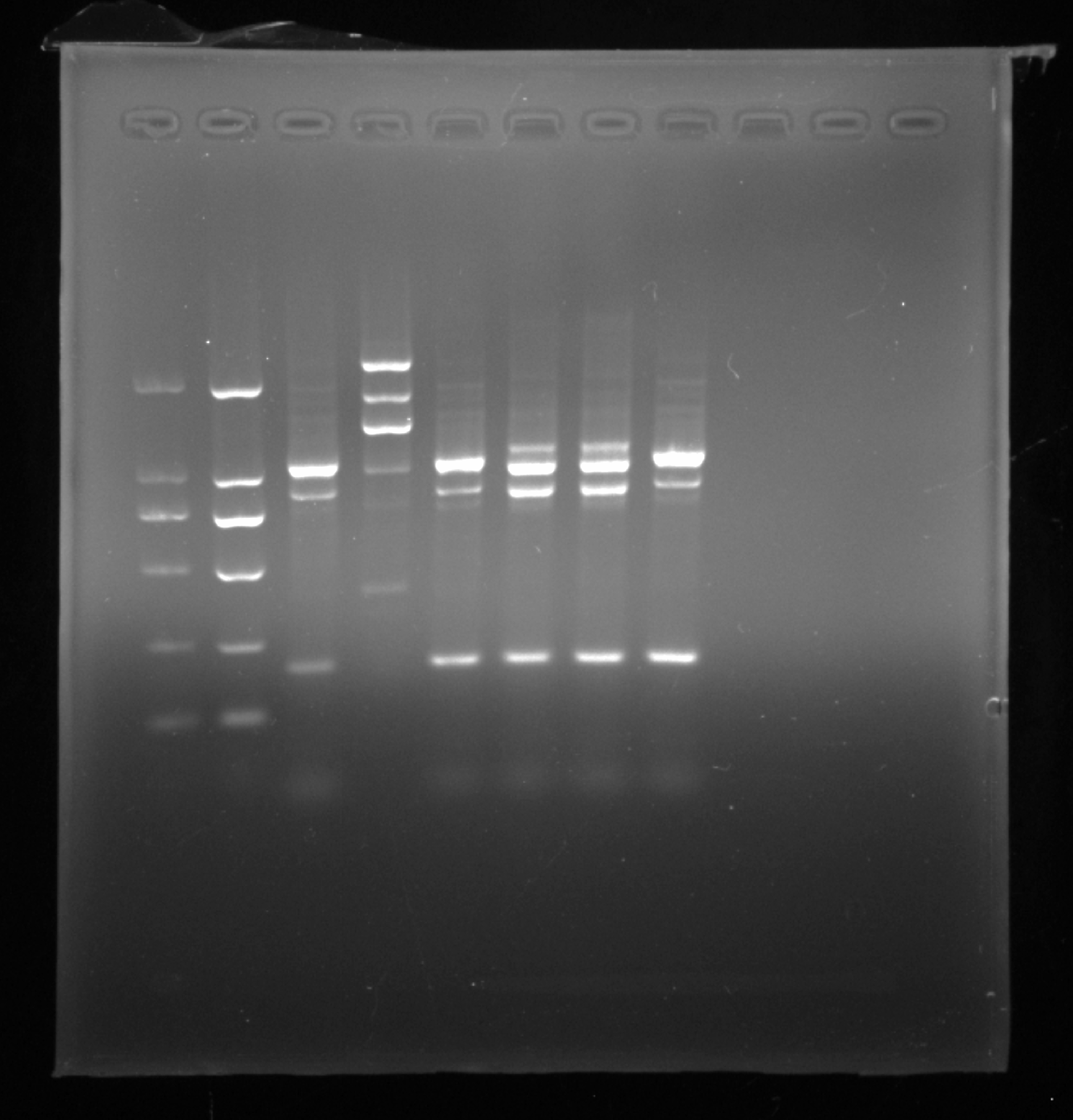


Figure S1. RT-PCR analysis of *MeFtsZ2-1* expression in OE plants using specific primers for the *MeFtsZ2-* gene. From left to right, lanes 1 and 2 are DNA standard markers; lanes 3-5 are RT-PCR results using WT DNA as a template; and lanes 6–8 are RT-PCR results using OE DNA as a template.


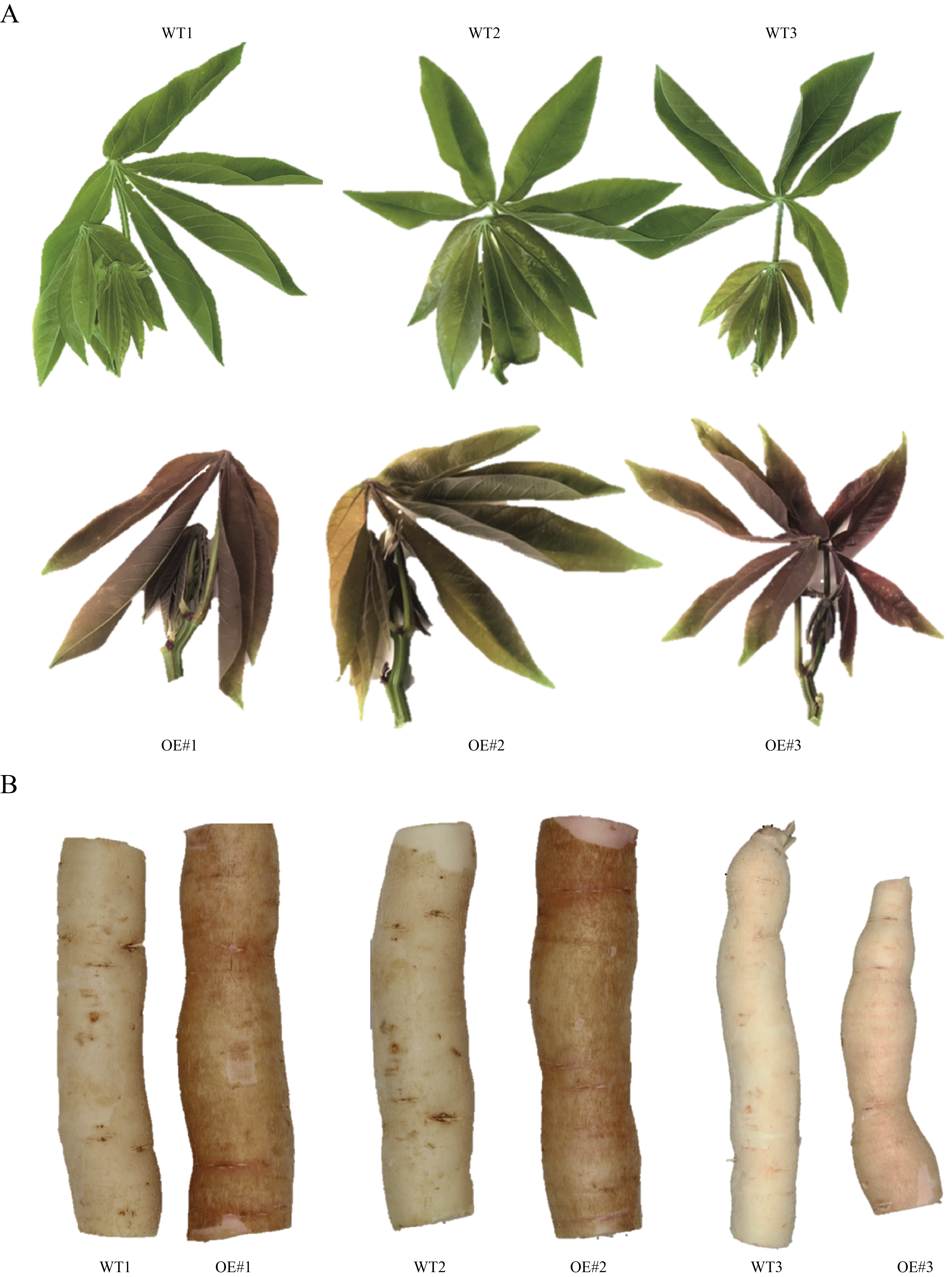


Figure S2. Leaf phenotype and tuberous root tuber phenotype. A. Leaf phenotype. B. tuberous root tuber phenotype. WT, wild-type cassava. OE#1, OE#2 and OE#3, *MeFtsZ2-1* overexpression cassava.


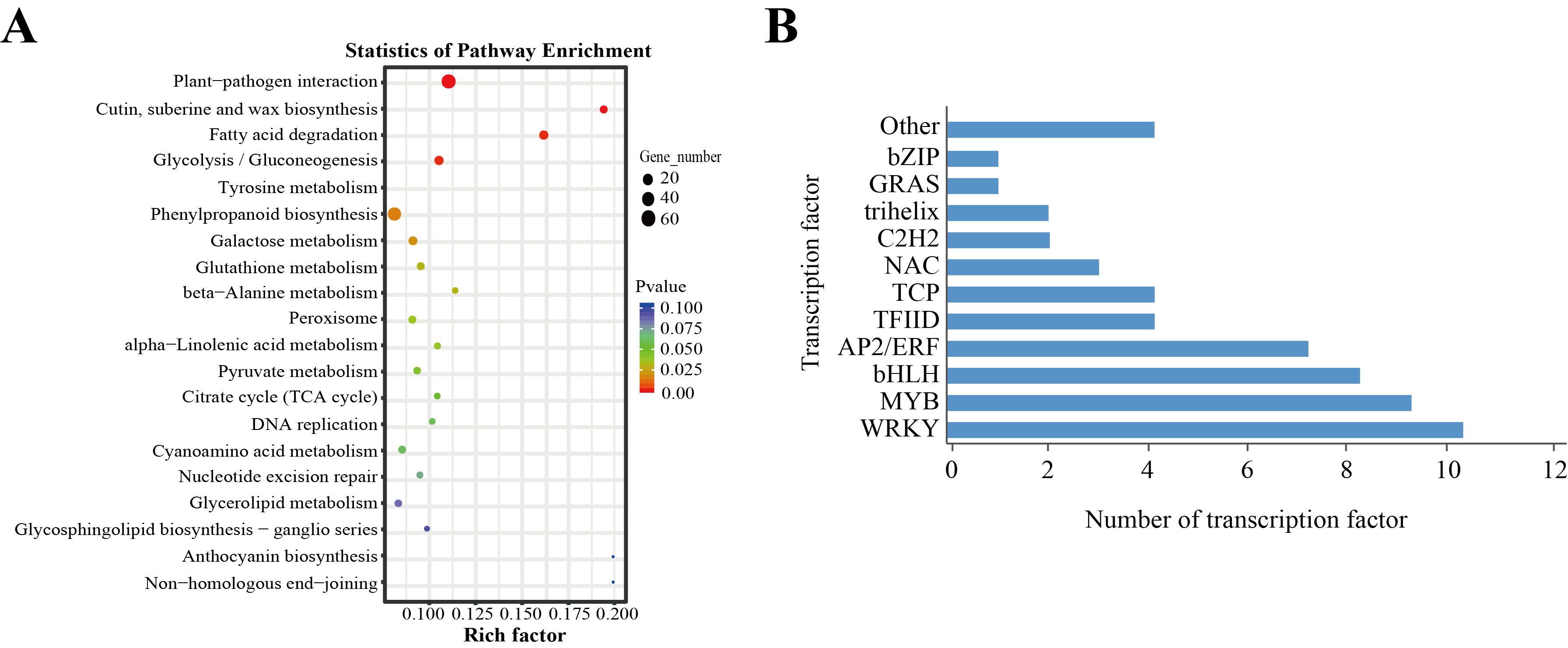


Figure S3. The KEGG enrichment analysis of the differentially expressed genes and transcription factor families between OE and WT. A. KEGG enrichment analysis of the DEGs in OEL/WTL. B. Transcription factor in OEL/WTL. OEL, *MeFtsZ2-1* overexpression cassava leaves. WTL, wild-type cassava leaves.


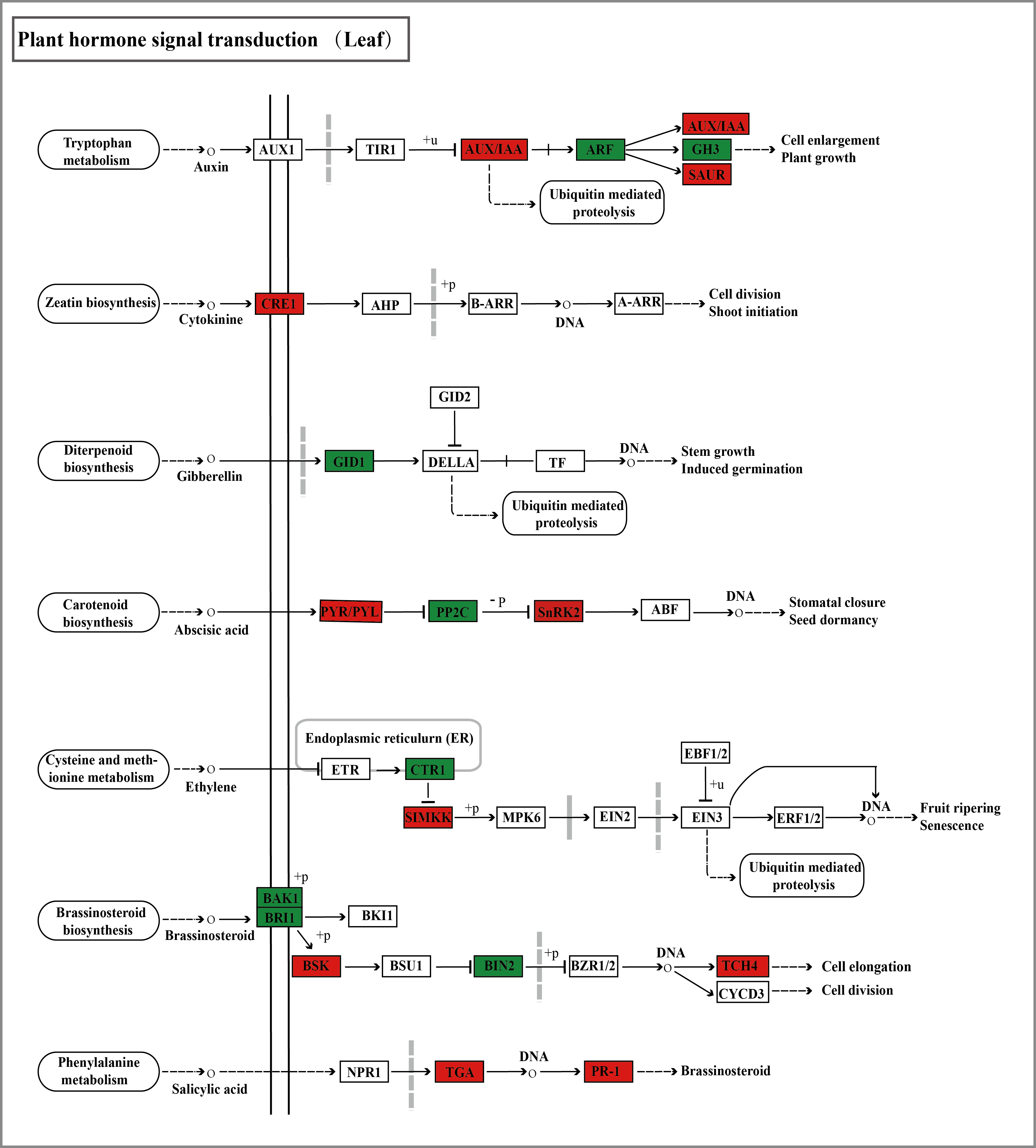


Figure S4. Changes in genes involved in the plant hormone signal transduction pathway in cassava leaves. Hormone signal transduction pathway in OEL/WTL. Green color indicates downregulated expression compared with WT, and red color indicates upregulated expression, yellow indicates both upregulated and downregulated. (For interpretation of the references to color in this figure legend, the reader is referred to the web version of this article.) OEL, *MeFtsZ2-1* overexpression cassava leaves. WTL, wild-type cassava leaves.
